# New Insights into the Mechanism of *Ulva pertusa* on Colitis in Mice: Modulation of the Pain and Immune System

**DOI:** 10.3390/md21050298

**Published:** 2023-05-13

**Authors:** Alessio Ardizzone, Deborah Mannino, Anna Paola Capra, Alberto Repici, Alessia Filippone, Emanuela Esposito, Michela Campolo

**Affiliations:** Department of Chemical, Biological, Pharmaceutical and Environmental Sciences, University of Messina, Viale Ferdinando Stagno d’Alcontres, 98166 Messina, Italy; aleardizzone@unime.it (A.A.); deborah.mannino@unime.it (D.M.); annapaola.capra@unime.it (A.P.C.); alberto.repici@studenti.unime.it (A.R.); alessia.filippone@unime.it (A.F.); campolom@unime.it (M.C.)

**Keywords:** inflammatory bowel diseases (IBDs), ulcerative colitis (UC), *Ulva pertusa*, abdominal pain, TLR4, NLRP3 inflammasome

## Abstract

Inflammatory bowel diseases (IBDs) involving Crohn’s disease (CD) and ulcerative colitis (UC) are gastrointestinal (GI) disorders in which abdominal pain, discomfort, and diarrhea are the major symptoms. The immune system plays an important role in the pathogenesis of IBD and, as indicated by several clinical studies, both innate and adaptative immune response has the faculty to induce gut inflammation in UC patients. An inappropriate mucosal immune response to normal intestinal constituents is a main feature of UC, thus leading to an imbalance in local pro- and anti-inflammatory species. *Ulva pertusa*, a marine green alga, is known for its important biological properties, which could represent a source of beneficial effects in various human pathologies. We have already demonstrated the anti-inflammatory, antioxidant, and antiapoptotic effects of an *Ulva pertusa* extract in a murine model of colitis. In this study, we aimed to examine thoroughly *Ulva pertusa* immunomodulatory and pain-relieving properties. Colitis was induced by using the DNBS model (4 mg in 100 μL of 50% ethanol), whereas *Ulva pertusa* was administered daily at the dosage of 50 and 100 mg/kg by oral gavage. *Ulva pertusa* treatments have been shown to relieve abdominal pain while modulating innate and adaptative immune-inflammatory responses. This powerful immunomodulatory activity was specifically linked with TLR4 and NLRP3 inflammasome modulation. In conclusion, our data suggest *Ulva pertusa* as a valid approach to counteract immune dysregulation and abdominal discomfort in IBD.

## 1. Introduction

Natural compounds, whose use has greatly increased in the last two decades, have proven highly effective in reducing the inflammatory state in several intestinal disorders including ulcerative colitis (UC), efficiently restoring intestinal homeostasis [1,2].

For instance, curcumin or melatonin have been shown to inhibit NLRP3 inflammasome and TLR4/MyD88/NF-κB signaling pathway respectively, reducing abdominal pain as well as acting like immune checkpoints in preclinical models of colitis [3,4,5].

Natural compounds provide the advantages of high efficacy and fewer adverse reactions compared to conventional drugs, thus representing a valid alternative treatment in the management of UC so as a promising tool to identify novel candidate drugs [6].

In light of this, marine ecosystem compounds could also represent an important source of biologically active molecules, thus supporting the development of innovative pharmacological strategies. In particular, *Ulva pertusa* is a green alga belonging to the Ulvaceae family and is widely used in traditional Chinese medicine thanks to its multiple biological properties [7].

We previously demonstrated the noteworthy pharmacological activities of an *Ulva pertusa* extract through the modulation of SIRT1/Nrf2/NF-κB signaling pathways in a murine model of colitis [8], which resulted in a favorable decrease in infiltrating immune cells such as mast cells and neutrophils in the intestinal environment. Those positive outcomes suggested a good immunomodulatory capacity of *Ulva pertusa* to protect the colon from UC-related immune dysregulation worthy of detailed investigation in future studies.

Certainly, the understanding of the immune pathogenetic mechanisms causing UC is useful in the development of new effective care. At the basis of this intestinal disease, long-lasting immune-inflammatory responses have been identified. In particular, these harmful mechanisms seem to be derived from an excessive immune response directed against microbial or environmental-derived antigens that can be triggered by the breakdown of the intestinal epithelial barrier integrity [9]. To this, clinical scientific reports suggested that both dysregulated innate and adaptive immune pathways contribute to abdominal pain and aberrant intestinal inflammatory response in UC patients [10,11]. The deepening of interactions between different components of the innate and adaptive immune systems, such as neutrophils, macrophages, and T cells could open new horizons in the knowledge about mechanisms of gut inflammation [12].

Overall, considering the involvement of both innate and adaptive immune systems in UC pathogenesis, the development of several immune-targeted treatments could constitute a valuable strategy for decreasing colitis features due to cytokine storms and immune system dysregulation in the colonic environment.

Based on these assumptions, here we aimed to deep the immunomodulatory abilities of *Ulva pertusa* extract, focusing on mechanisms of action not yet identified and evaluating pain-relieving activities, by using a mouse model of DNBS-induced colitis.

## 2. Results

### 2.1. Effect of Ulva pertusa Extract on Body Weight, Colon Length, and Histological Evaluations

Body weight was assessed throughout the experiment to determine whether DNBS-induced colitis had occurred successfully. In accordance, in DNBS+vehicle mice we detected a significant reduction in body weight compared to the Sham group (see Figure S1). However, oral administration of *Ulva pertusa* showed beneficial effects by reducing body weight loss in treated mice, particularly at the dose of 100 mg/kg (see Figure S1). Additionally, colonic shortening was significantly reduced by *Ulva pertusa* treatments, thus denoting a good control of colonic inflammation (see Figure S1), whereas no significant changes were found between the Sham+vehicle group and the Sham+*Ulva pertusa* treated mice in all parameters analyzed (body weight, colon length, and histological score; see Figure S1).

### 2.2. Ulva pertusa Extract Relieved DNBS-Induced Visceral Hyperalgesia and Visceral Hypersensitivity

**Figure 1:**
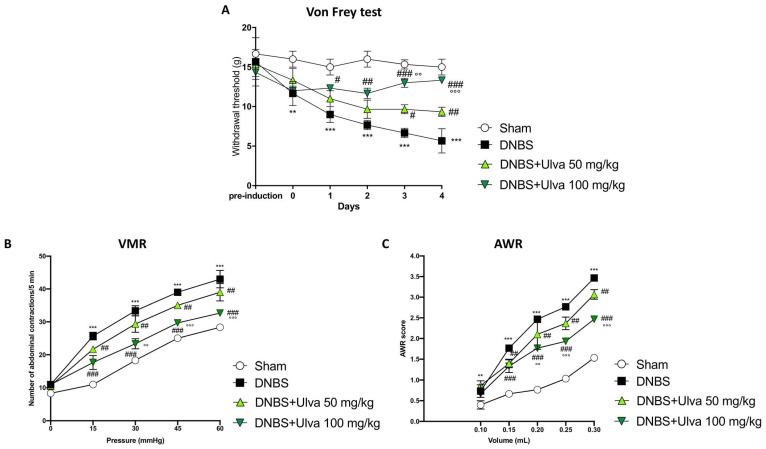
Pain-relieving properties of *Ulva pertusa* extract. Following DNBS-induced colitis and treatment with *Ulva pertusa* at doses of 50 mg/kg and 100 mg/kg, the Von Frey test was used to measure the withdrawal threshold as an indicator of abdominal pain over a 5-day period (**A**). In addition, two tests in 5-day period were used to assess the pain: Visceromotor Response (**B**) and Abdominal Withdrawal Reflex (**C**). In every experimental group the number of mice was n = 10. Values are means ± SD. The two-way ANOVA test was followed by the Bonferroni test. ** 0.01 vs. Sham; *** *p* < 0.001 vs. Sham; # *p* < 0.05 vs. DNBS; ## *p* < 0.01 vs. DNBS; ### *p* < 0.001 vs. DNBS; °° *p* < 0.01 vs. DNBS+Ulva 50 mg/kg; °°° *p* < 0.001 vs. DNBS+Ulva 50 mg/kg.

Cytokines and inflammatory mediators enhance the excitability of sensory nerves that carry information from the gut to the brain. For these reasons, inflammation associated with colitis is considered a major cause of abdominal pain [13,14]. To evaluate the effects of *Ulva pertusa* extract in reducing abdominal pain, the von Frey test was performed. Harmless mechanical stimulation of the abdomen (a measure similar to allodynia) showed a reduced withdrawal response in animals subjected to intrarectal injection of DNBS up to day 5, compared to Sham animals (Figure 1A). *Ulva pertusa* administration (50–100 mg/kg) in a dose-dependent manner increased the pain threshold, in particular, the highest dose of 100 mg/kg was significantly effective in reducing DNBS-induced hyperalgesia, especially after 4 days of treatment (Figure 1A). Visceral sensitivity was assessed by measuring the visceromotor response (VMR) and abdominal withdrawal reflex (AWR) to colorectal distension (CRD). Five days after DNBS injection, both VMR and AWR were significantly higher than in the Sham group (Figure 1B,C). Oral administration of *Ulva pertusa* at doses of 50 mg/kg and 100 mg/kg reduced DNBS-induced visceral hypersensitivity (Figure 1B,C). Particularly, the higher dose of 100 mg/kg demonstrated greater efficacy in reducing abdominal pain than the 50 mg/kg dose.

### 2.3. Ulva pertusa Extract Reduced Colonic Damage and Neutrophilic Infiltration Induced by DNBS Instillation

**Figure 2:**
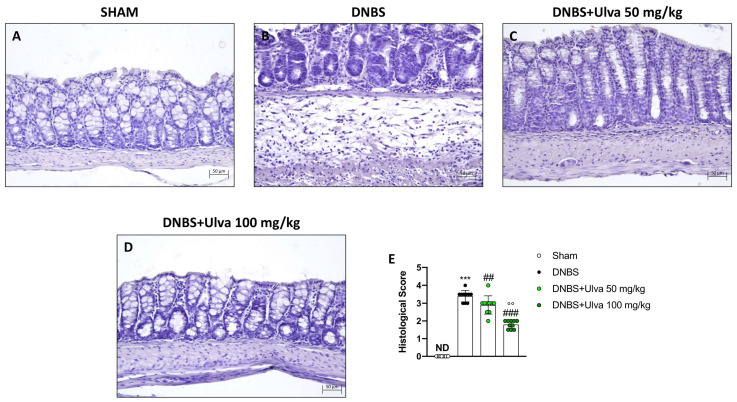
Effect of *Ulva pertusa* extract on colonic histological damage induced by DNBS. Hematoxylin and Eosin staining showed tissue damage in mice treated only with DNBS ((**B**), score (**E**)) when compared with the control group ((**A**), score (**E**)). *Ulva pertusa* 50 mg/kg ((**C**), score (**E**)) and 100 mg/kg ((**D**), score (**E**)) reversed colitis features. The results of the histological examination were displayed at 20× magnification. In every experimental group the number of mice was n = 10. Values are means ± SD. The one-way ANOVA test was followed by the Bonferroni test. *** *p* < 0.001 vs. Sham; ## *p* < 0.01 vs. DNBS; ### *p* < 0.001 vs. DNBS; °° *p* < 0.01 vs. DNBS+Ulva 50 mg/kg.

To confirm the success of the DNBS-induced colitis model and the protective effect of *Ulva pertusa* in counteracting DNBS-induced colitis, as reported in our previous study, colon sections were stained with Hematoxylin and Eosin (H&E). In this new set of experiments, histological results established that rectal instillation of DNBS induced ulcerative colitis in mice by causing histopathological changes in colonic mucosa, also reconfirming *Ulva pertusa* skills in reversing colonic tissue damage. Indeed, DNBS-treated animals showed histopathological changes characterized by loss of crypt architecture, tissue edema, and a large number of neutrophils in the submucosa layer (Figure 2B,E). Furthermore, in agreement with what was previously demonstrated, oral treatment with *Ulva pertusa* significantly improved tissue architecture (Figure 2C,E), especially at the higher dose of 100 mg/kg (Figure 2D,E).

### 2.4. Ulva pertusa Extract Reduced Intercellular Adhesion Molecule (ICAM)-1 and p-Selectin Expression and Serum Cytokine Levels following DNBS-Induced Colitis

**Figure 3:**
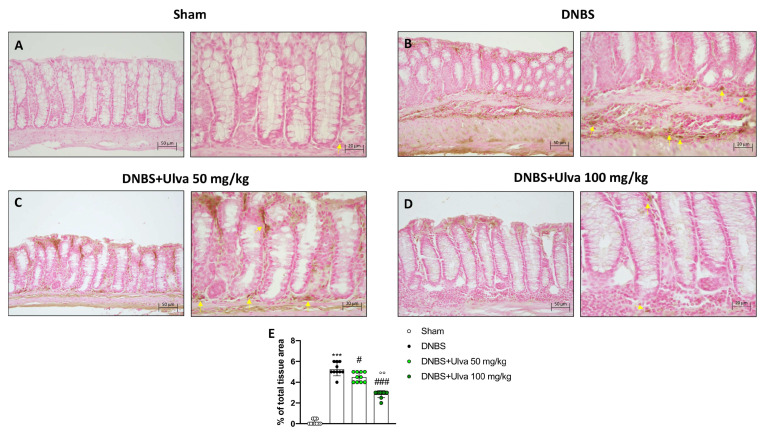
Effect of *Ulva pertusa* administration on ICAM expression. Immunohistochemical analyses was performed to evaluate ICAM-1 expression in the Sham group ((**A**), 20× **left** and 40× **right**, score (**E**)), DNBS-injected animals ((**B**), 20× **left** and 40× **right**, score (**E**)), and mice treated with *Ulva pertusa* at both doses of 50 mg/kg ((**C**), 20× **left** and 40× **right**, score (**E**)) and 100 mg/kg ((**D**), 20× **left** and 40× **right**. score (**E**)). Yellow arrows indicate the positive staining for ICAM-1. In every experimental group the number of mice was n = 10. The results of the immunohistochemical staining were displayed at 20× and 40× magnification. Values are means ± SD. The one-way ANOVA test was followed by the Bonferroni test. *** *p* < 0.001 vs. Sham; # *p* < 0.05 vs. DNBS; ### *p* < 0.001 vs. DNBS: °° *p* < 0.01 vs. DNBS+Ulva 50 mg/kg.

**Figure 4:**
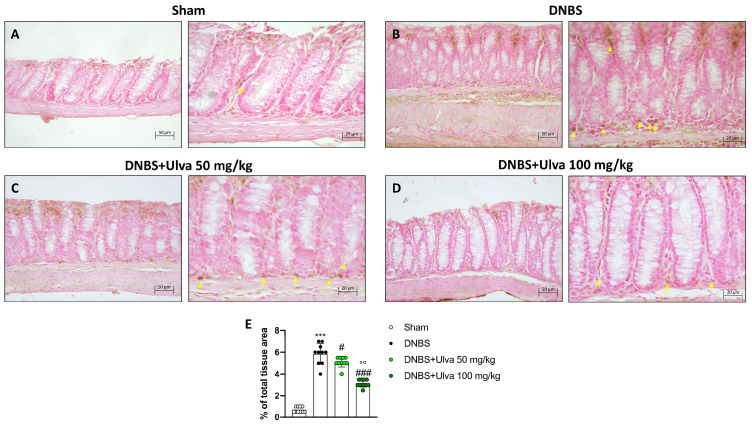
Effect of *Ulva pertusa* administration on p-Selectin expression. Positive p-Selectin immunostaining was found in colon tissues collected from DNBS+vehicle mice ((**B**), 20× left and 40× right, score (**E**)), compared to the Sham animals ((**A**), 20× **left** and 40× **right**, score (**E**)). *Ulva pertusa* treatment at the dose of 50 mg/kg is shown ((**C**), 20× **left** and 40× **right**, score (**E**)) and 100 mg/kg is also reported ((**D**), 20× **left** and 40× **right**, score (**E**)) reduced positive staining. Yellow arrows indicate the positive staining for p-Selectin. In every experimental group the number of mice was n = 10. The results of the immunohistochemical staining were displayed at 20× and 40× magnification. Values are means ± SD. he one-W=way ANOVA test was followed by the Bonferroni test. *** *p* < 0.001 vs. Sham; # *p* < 0.05 vs. DNBS; ### *p* < 0.001 vs. DNBS; °° *p* < 0.01 vs. DNBS+Ulva 50 mg/kg.

The infiltration of leukocytes from the bloodstream into the intestinal tissue, responsible for the inflammatory process in IBD, is mediated by a family of membrane molecules, called cell adhesion molecules (CAMs). In IBD, ICAM-1, and p-Selectin are the CAMs mainly involved in cell recruitment [15]. Our data demonstrated that positive staining for ICAM-1 and p-Selectin was markedly increased in colonic tissues from DNBS-injected mice (Figure 3B,E and Figure 4B,E) compared to Sham mice (Figure 3A,E and Figure 4A,E). Oral treatment with *Ulva pertusa* was able to significantly reduce immunopositive staining for ICAM-1 and P-selectin (Figure 3 3C,E and Figure 4C,E), mainly at the dose of 100 mg/kg (Figure 3D,E and Figure 4D,E).

**Figure 5:**
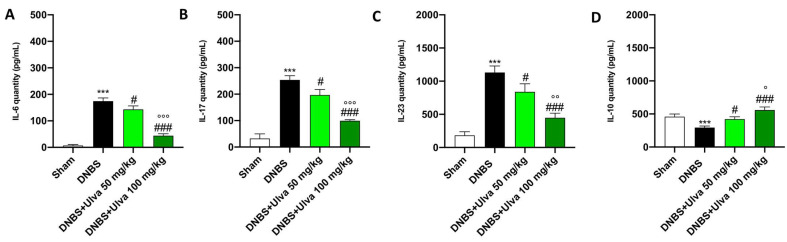
*Ulva pertusa* administration modulated interleukins after DNBS-induced colitis. Serum levels of the following interleukins have been assessed by ELISA kit: IL-6 (**A**), IL-17 (**B**), IL-23 (**C**), IL-10 (**D**). In every experimental group the number of mice was n = 10. Values are means ± SD. The one-way ANOVA test was followed by the Bonferroni test. *** *p* < 0.001 vs. Sham; # *p* < 0.05 vs. DNBS; ### *p* < 0.001 vs. DNBS; ° *p* < 0.05 vs. DNBS+Ulva 50 mg/kg; °° *p* < 0.01 vs. DNBS+Ulva 50 mg/kg; °°° *p* < 0.001 vs. DNBS+Ulva 50 mg/kg.

Furthermore, CAMs through the recruitment of immune cells contribute to the overproduction of proinflammatory cytokines, which were measured by enzyme-linked immunosorbent assay (ELISA). In this context, our study demonstrated that DNBS-injected mice had elevated serum levels of pro-inflammatory cytokines such as IL-6, IL-17, and IL-23 and reduced levels of the anti-inflammatory cytokine IL-10 (Figure 5A–D). Oral treatments with *Ulva pertusa*, especially at the higher dose of 100mg/kg, significantly reduced serum IL-6, IL-17, and IL-23 levels and enhanced serum IL-10 levels (Figure 5A–D).

### 2.5. Ulva pertusa Extract Treatment Markedly Reduced CD68^+^ Macrophages Marker following DNBS-Induced Colitis

The innate immune response in IBD is also mediated by macrophages which, through antigens presentation to specific lymphocytes, leads to the activation of adaptive immune cells [16].

Thus, immunofluorescence analysis was performed to evaluate the number of CD68^+^ cells, a lysosomal membrane receptor highly expressed in macrophages.

**Figure 6:**
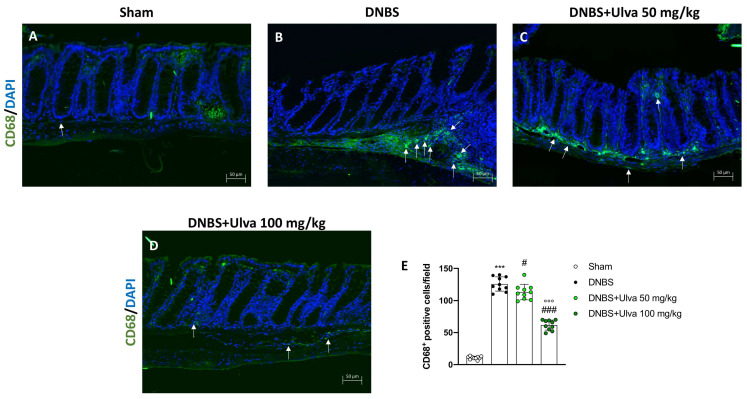
Effect of *Ulva pertusa* administration on CD68 expression. To assess the amount of CD68^+^ cells immunofluorescence was performed: Sham group ((**A**), score (**E**)), group with DNBS-induced colitis ((**B**), score (**E**)), treatment with *Ulva pertusa* 50 mg/kg ((**C**), score (**E**)) and *Ulva pertusa* 100 mg/kg ((**D**), score (**E**)). White arrows indicate the positive staining for CD68^+^. The results of the immunofluorescence staining were displayed at 20× magnification. In every experimental group the number of mice was n = 10. Values are means ± SD. The one-way ANOVA test was followed by the Bonferroni test. *** *p* < 0.001 vs. Sham; # *p* < 0.05 vs. DNBS; ### *p* < 0.001 vs. DNBS; °°° *p* < 0.001 vs. DNBS+Ulva 50 mg/kg.

Five days after the intrarectal injection of DNBS, a significant increase in the number of CD68^+^ cells were observed in the colon sections (Figure 6B,E) compared to Sham animals’ tissues (Figure 6A,E). Treatment with *Ulva pertusa* was able to significantly reduce the number of positive cells, especially at the highest dose of 100 mg/kg (Figure 6C,D,E).

### 2.6. Ulva pertusa Extract Modulated the Number of Cluster of Differentiation (CD)4^+^ and CD8^+^ Antigens following DNBS-Induced Colitis

**Figure 7:**
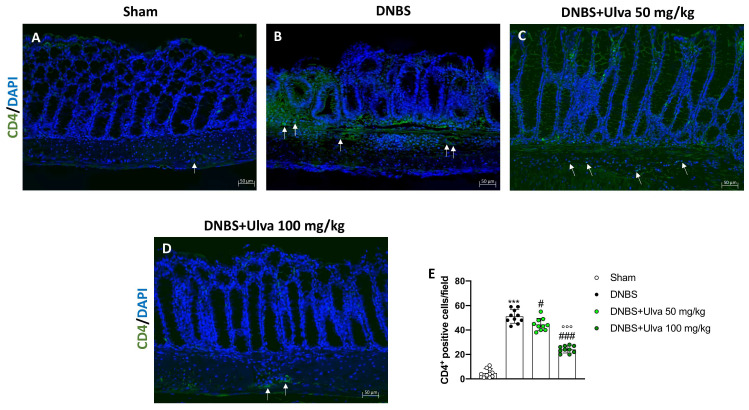
Effect of *Ulva pertusa* administration on CD4 expression. Immunofluorescence analysis of CD4 in the colon tissues of Sham group ((**A**), score (**E**)), DNBS-injected animals ((**B**), score (**E**)), mice treated with *Ulva pertusa* 50 mg/kg ((**C**), score (**E**)) and *Ulva pertusa* 100 mg/kg ((**D**), score (**E**)). White arrows indicate the positive staining for CD4. The results of the immunofluorescence staining were displayed at 20× magnification. In every experimental group the number of mice was n = 10. Values are means ± SD. The one-way ANOVA test was followed by the Bonferroni test. *** *p* < 0.001 vs. Sham; # *p* < 0.05 vs. DNBS; ### *p* < 0.001 vs. DNBS; °°° *p* < 0.001 vs. DNBS+Ulva 50 mg/kg.

To study the effects of *Ulva pertusa* in modulating adaptive immune response we evaluated the number of CD4^+^ and CD8^+^ T-positive cells. Immunofluorescence results demonstrated that the number of CD4^+^ T-positive cells was significantly reduced in colonic sections of *Ulva pertusa*-treated mice at doses of 50 mg/kg and 100 mg/kg (Figure 7C,D,E) compared with DNBS-injected mice (Figure 7B,E). Sham mice showed fewer CD4^+^ T-positive cells (Figure 7A,E).

**Figure 8:**
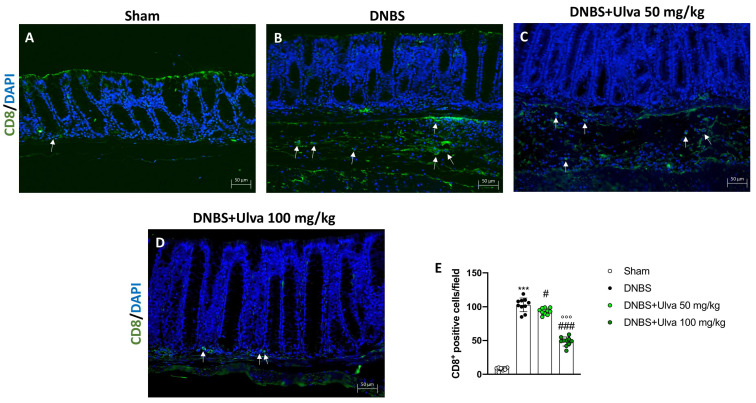
Effect of *Ulva pertusa* administration on CD8 expression. Colon sections were stained with immunofluorescence to evaluate the amount of CD8 in Sham group ((**A**), score (**E**)), DNBS-injected animals ((**B**), score (**E**)), treatment with *Ulva pertusa* 50 mg/kg ((**C**), score (**E**)) and *Ulva pertusa* 100 mg/kg ((**D**), score (**E**)). White arrows indicate the positive staining for CD8. The results of the immunofluorescence staining were displayed at 20× magnification. In every experimental group the number of mice was n = 10. Values are means ± SD. The one-way ANOVA test was followed by the Bonferroni test. *** *p* < 0.001 vs. Sham; # *p* < 0.05 vs. DNBS; ### *p* < 0.001 vs. DNBS; °°° *p* < 0.001 vs. DNBS+Ulva 50 mg/kg.

Likewise, DNBS-injected mice showed a high number of CD8^+^ T-positive cells (Figure 8B,E) if compared to Sham animals (Figure 8A,E). Differently, mice treated with *Ulva pertusa* revealed a significantly reduced number of CD8^+^ T-positive cells in a dose-dependent manner (Figure 8C,D,E), thus demonstrating a modulatory effect on T cell adaptive immunity.

### 2.7. Ulva pertusa Treatments Modulated TLR4/Myd88/TRAF6 Pathway and NLPR3 Inflammasome following DNBS-Induced Colitis

Since activation of TLR4/Myd88/TRAF6 signaling triggers the downstream cascades involved in IBD pathogenesis and that the TLR4 receptor possesses a high affinity for many exogenous ligands of natural origin [17], based on previous results, we hypothesized that the modulation of immuno-inflammatory pathways by *Ulva pertusa* could be related to TLR4/Myd88-dependent signaling downregulation.

**Figure 9:**
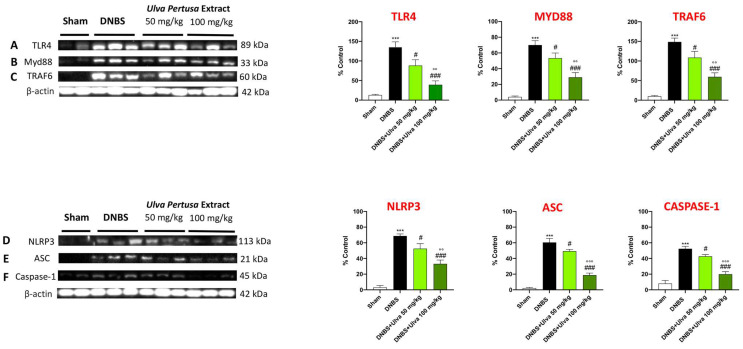
Effect of *Ulva pertusa* on TLR4 and NLRP3 signaling pathways. Western Blot results of: TLR4 (**A**), Myd-88 (**B**), TRAF6 (**C**), NLRP3 (**D**), ASC (**E**), Caspase-1 (**F**). In every experimental group the number of mice was n = 10. Values are means ± SD. The one-way ANOVA test was followed by the Bonferroni test. *** *p* < 0.001 vs. Sham; # *p* < 0.05 vs. DNBS; ### *p* < 0.001 vs. DNBS; °° *p* < 0.01 vs. DNBS+Ulva 50 mg/kg; °°° *p* < 0.001 vs. DNBS+Ulva 50 mg/kg.

For this purpose, we assessed TLR4, Myd88, and TRAF6 expression in colonic tissues by Western Blot analysis. The obtained results demonstrated that TLR4, Myd88, and TRAF6 expression was significantly upregulated in colon samples after intrarectal DNBS treatment compared to the Sham group (Figure 9A–C). Unlike DNBS-injected mice, proteins expression in the colon of *Ulva pertusa*-treated mice were significantly downregulated in a dose-dependent manner (Figure 9A–C,). In response to TLR4 activation, the signaling molecule MyD88 regulates the induction of NLRP3. NLRP3 combines with the ASC adapter and induces pro-Caspase-1 translocation and activation thereby contributing to IBD through the induction of immune responses [18,19]. For this reason, we investigated the effects of *Ulva pertusa* in the modulation of the NLRP3/ASC/Caspase-1 pathway. Western Blot analysis showed an evident increase of NLPR3, ASC, and Caspase-1 protein expressions in colon tissues of DNBS-injected mice compared to Sham mice (Figure 9D–F,). However, the expression of NLPR3, ASC, and Caspase-1 proteins were significantly reduced in *Ulva pertusa*-treated mice, especially at the higher dose of 100 mg/kg (Figure 9D–F).

### 2.8. Protective Role of Ulva pertusa in Restoring Goblet Cells Impaired by TLR4 Signaling after DNBS-Induced Colitis

The activation of the TLRs/Myd88 pathway triggers nonspecific endocytosis of goblet cells which are killed and expelled into the lumen resulting in reduced transepithelial mucin secretion [20].

To learn more about the protective role of *Ulva pertusa*, PAS staining was performed to identify the number of goblet cells in colon samples.

**Figure 10:**
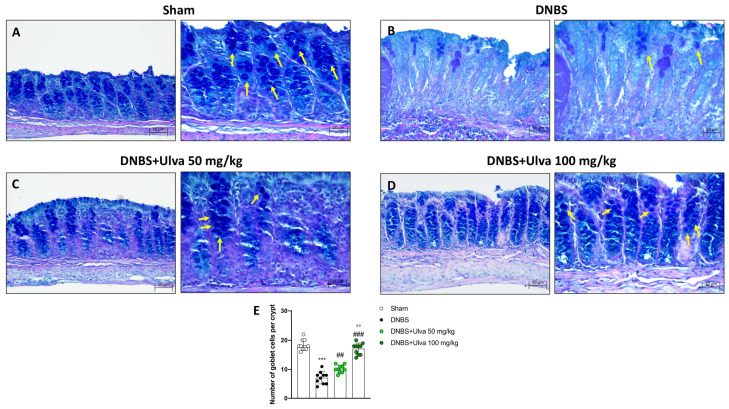
Effect of *Ulva pertusa* administration on goblet cells number. The Alcian Blue/Pas staining was conducted on colon tissues for the evaluation of the globet cells: Sham group ((**A**), 20× **left** and 40× **right**, score (**E**)), DNBS-injured mice ((**B**), 20× **left** and 40× **right**, score (**E**)), *Ulva pertusa* 50 mg/kg ((**C**), 20× **left** and 40× **right**, score (**E**)) and *Ulva pertusa* 100 mg/kg ((**D**), 20× **left** and 40× **right**, score (**E**)). The results of the Alcian Blue/Pas staining were displayed at 20× and 40× magnification. Yellow arrows indicate goblet cells. In every experimental group the number of mice was n = 10. Values are means ± SD. The one-way ANOVA test was followed by the Bonferroni test. *** *p* < 0.001 vs. Sham; ## *p* < 0.01 vs. DNBS; ### *p* < 0.001 vs. DNBS; °° *p* < 0.01 vs. DNBS+Ulva 50 mg/kg.

Our results revealed that compared to Sham mice (Figure 10A,E), the number of goblet cells in the colonic crypts of DNBS-injected mice was markedly reduced (Figure 10B,E). In contrast, goblet cells showed almost complete recovery in colonic sections of mice treated with *Ulva pertusa* 50 mg/kg and 100 mg/kg (Figure 10C,E and Figure 10D,E, respectively). Thus, as the TLR4 pathway impairs goblet cells in IBD, these findings confirm a protective role of *Ulva pertusa* that may be closely related to TLR4 signaling.

## 3. Discussion

UC is a highly debilitating gastrointestinal disease belonging to the large and heterogeneous family of IBDs [21]. The disease is characterized by a relapsing-remitting course, with symptoms that include mostly gastrointestinal signs but sometimes also neurological or cutaneous complications [22,23]. Several pharmacological agents have been proposed over the years;, however, although these drugs have proved capable of decreasing the symptoms of UC-affected patients, none of these treatments has proved pivotal for the resolution of the pathology [24]. Thus, the lack of effective therapies has prompted researchers to investigate more deeply the pathogenetic mechanisms underlying UC in order to discover attractive biological targets as well as the most effective treatments, with both synthetic or natural compounds.

The present study aimed to further investigate the pharmacological properties of an *Ulva pertusa* extract, especially focusing on pain-relievers, and immunomodulatory benefits from this precious macroalgae would be extremely useful for UC patients in whom abdominal discomfort as well as immune system dysregulation are characteristic features [25].

More than 50% of UC individuals experienced abdominal discomfort, which negatively affects patients’ daily activities and considerably impacts their quality of life [26]. Our study showed a substantial visceral hypersensitivity caused by the DNBS administration, resulting in excessive abdominal pain and colon responsiveness. However, the administration of *Ulva pertusa* extract, in a dose-dependent manner, counteracted the increase of visceral sensitivity caused by DNBS injection, highlighting a powerful analgesic action to moderate abdominal pain triggered by the inflammatory process in the colon.

The inflammatory pathway and immune response analyzed in UC patients have shown that tissue damage is driven by complex networks and biological crosstalk of immune cells and cytokines [27].

Indeed, numerous studies indicated that the gut environment is constantly monitored by the host mucosal immune system, and any slight disturbance in the microbial communities may contribute to intestinal immune disruption thus increasing the susceptibility to the establishment and development of IBDs [28].

Furthermore, in a genetically susceptible host, CAMs through complex interactions contribute to the recruitment of an immunologically rich cellular population in the intestinal mucosa layer, triggering local disruption of the gut barrier epithelium in IBDs [29]. Such dysregulation of innate and adaptive intestinal immune responses, cooperatively to the enrollment of inflammatory mediators operated by CAMs, increases the trafficking and migration of further immune cells like macrophages towards the intestinal site [30]. Overall, after DNBS–intrarectal injection, we found an uncontrolled T cell activation in intestinal mucosa together with an elevated macrophage infiltration promoted by important adhesion molecules such as ICAM and p-Selectin. *Ulva pertusa* treatments, in a dose-dependent manner, significantly decreased the expression of the CAMs like ICAM and p-Selectin as well as CD4^+^ and CD8^+^ T lymphocytes in colon tissue while reducing CD68^+^ positive cells, a marker identifying macrophages.

Moreover, the reverse of immune-inflammatory responses exerted by *Ulva pertusa* resulted in a significant decrease of proinflammatory serum biomarkers such as IL-6, IL-17, and IL-23, and in a favorable restoration of the anti-inflammatory IL-10.

Considering the noteworthy ability of *Ulva pertusa* in modulating immune cells in the colonic mucosa, we deepened the mechanism underlying such immunomodulatory action. In this regard, it is known that TLRs are the best-characterized transmembrane receptors present in various intestinal cells, possessing the ability to interact with the gut microbiota so as to mediate inflammatory immune responses and maintain intestinal epithelial homeostasis through MyD88 signaling [28]. Indeed, the overactivation of TLRs-dependent immune mechanisms can trigger an excessive inflammatory reaction favoring the onset of autoimmune-related diseases such as colitis [31,32]. In this regard, TLR4 hyperactivation emerged as the major orchestrator for the development of the inflammatory reaction in colitis, compromising the regeneration of the intestinal mucosa and increasing over time the risk of developing inflammation-associated colon cancer [33]. Therefore, we speculated that the immunomodulatory properties of *Ulva pertusa* could be related to the modulation of TLR4, thus advancing the hypothesis that *Ulva pertusa* could act as a receptor antagonist. The obtained results confirmed our theory; we found a marked activation of the TLR4-MyD88 signaling pathway following DNBS-induced colitis. Differently, *Ulva pertusa* daily treatment showed positive outcomes by modulating TLR4 as well as Myd88 and TRAF6 expressions, denoting a good immunomodulatory capacity to protect the colon from UC immune alterations.

Compelling evidence demonstrated that the secretion of pro-inflammatory cytokines and interleukins is shaped by multiple inflammation signaling pathways, especially by the TLR4 and NLRP3 inflammasome [34]. Indeed, at this crossroads of immune and inflammatory reactions, the NLRP3 inflammasome complex arises as a major component in exacerbating mucosal inflammation in UC [35]. Aberrant NLRP3 inflammasome activation involves the participation of varied components including ASC and Caspase-1 which contribute to the development of IBDs, including colitis [36]. Thus, regulating the TLR4-NLRP3 signaling pathway may be an encouraging therapeutic strategy to improve intestinal health. In the present study, we demonstrated an important activation of the NLRP3 complex following DNBS-induced colitis; however, *Ulva pertusa* treatments significantly decreased NLRP3, ASC, and Caspase-1 expressions, lessening NLRP3 inflammasome activity.

Recently, it has been discovered that bacterial TLRs ligand activating Myd88 promote non-specific endocytosis of goblet cells through their expulsion from the intestinal lumen [20]. This mechanism, although primarily aimed at protecting the gut epithelium from pathogens, leads in the long-term to a decrease of goblet cell number and consequently in a reduction of the protective mucus layer in the intestinal lumen. Accordingly, following colitis induction, our results confirmed a considerable decrease of goblet cells in colonic crypts, which was considerably reversed by the *Ulva pertusa* administration.

All these data have highlighted the benefits deriving from the administration of *Ulva pertusa*, which translate into the remodeling of the intestinal immune environment, thus avoiding prolonged immunoinflammatory reactions harmful to visceral hypersensitivity and intestinal homeostasis.

## 4. Materials and Methods

### 4.1. Materials

All the chemicals used in this study were of the highest commercial grade available, unless otherwise stated all the compounds used were obtained from Sigma–Aldrich (Milan, Italy). All the stock solutions were prepared in non-pyrogenic saline (0.9% NaCl; Baxter, Liverpool, UK). *Ulva pertusa* extract was a generous gift of the Chi.Bio.Far.Am. Department of the University of Messina (Messina, Italy).

### 4.2. Animals

Male CD1 mice (4 weeks old, body weight between 25–30 g), supplied by Envigo (Milan, Italy) were used. The animals were located in a controlled environment (22 ± 2 °C, 55 ± 15% relative humidity, 12 h light/dark cycle), with food and water ad libitum. We also checked the animal’s conditions daily for one week before the start of the study. This animal study was performed following Italian regulations on the use of animals (D.M.116192) and Directive legislation (EU) (2010/63/EU) amended by Regulation (EU) 2019/1010 as well as ARRIVE guidelines.

### 4.3. Induction of Experimental Colitis

Colitis was induced by one singular intrarectal administration with a low dose of DNBS (4 mg for each animal). This dose was established based on previous works, which showed how this dosage allows induction of colitis without being toxic [8]. The mice were anesthetized using Enflurane and immediately after 2,4,6-dinitrobenzene sulphonic acid (DNBS; 4 mg in 100 μL of 50% ethanol) was injected into the rectum through a catheter inserted 4.5 cm proximally to the anus, whereas Sham groups received a single intracolonic administration of saline.

### 4.4. Experimental Groups

Mice were randomly distributed among the following groups:

Group 1: Sham+vehicle: vehicle solution (saline) was administered by oral gavage for 4 days (N = 10).

Group 2: Sham+*Ulva pertusa* 50 mg/kg: *Ulva pertusa* extract 50 mg/kg was administered by oral gavage for 4 days (N = 10).

Group 3: Sham+*Ulva pertusa* 100 mg/kg: *Ulva pertusa* extract 100 mg/kg was administered by oral gavage for 4 days (N = 10).

Group 4: DNBS+vehicle: mice subjected to DNBS-colitis induction, then administered with vehicle solution (saline) by oral gavage every 24 h for 4 days, starting from 3 h after DNBS instillation (N = 10).

Group 5: DNBS+*Ulva pertusa* 50 mg/kg: mice subjected to DNBS-colitis induction, then administered with *Ulva pertusa* extract 50 mg/kg by oral gavage every 24 h for 4 days, starting from 3 h after the DNBS instillation (N = 10).

Group 6: DNBS+*Ulva pertusa* 100 mg/kg: mice subjected to DNBS-colitis induction, then administered with *Ulva pertusa* extract 100 mg/kg by oral gavage every 24 h for 4 days, starting from 3 h after the DNBS instillation (N = 10).

*Ulva pertusa* extract was water-soluble, and it was administered by oral gavage after dissolution in the saline solution. The doses used were selected according to previous experiments [8]. No significant changes were detected between Sham+vehicle mice and Sham+*Ulva pertusa* 50 mg/kg or Sham+*Ulva pertusa* 100 mg/kg groups (see Figure S1), therefore, in the subsequent analyses we reported only the Sham+vehicle group data. At the end of the experiment, mice were sacrificed, and the colons were surgically removed and cleaned. The samples were subsequently used for histological and biochemical analyses.

**Figure 11:**
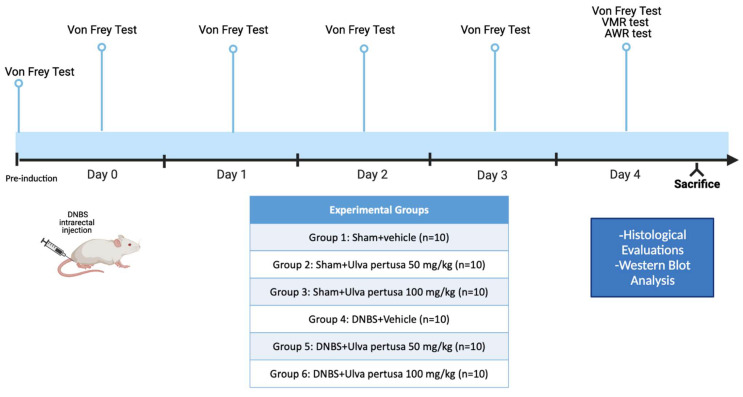
Graphical representation of the experimental design.

Timeline of the experiment was illustrated in Figure 11.

### 4.5. Von Frey, VMR, and AWR Tests

At the end of the experiment, the sensitivity and visceral pain caused by colitis were evaluated using three different methodologies such as Von Frey, VMR, and AWR tests. We followed all the steps based on previous studies [37,38,39] and they are briefly described below. Hypersensitivity to abdominal pain has been evaluated by the von Frey test, which consists of a force detector equipped with a plastic tip [38]. In short, mice were placed in plastic boxes under which there was a metallic net and were allowed to acclimate for 15 min. For the test, the tip was approached perpendicularly to the abdomen of each animal until a physical reaction, such as displacement and jumping, was observed. The mechanical threshold that represents the pressure that induces the behavioral reaction (the retraction of the abdomen) was immediately recorded by an electronic sensor to which the plastic tip was connected. The pressure was reproduced three times and then the average was performed for each animal [38]. The VMR test allowed us to assess pain sensitivity, and after the general anesthesia, two electromyography (EMG) electrodes were placed on the oblique muscle of the abdomen of each animal [39]. For this test, an oiled plastic balloon was used, inserted about 6.5 cm from the anus, with a catheter connected to a sterile syringe with increasing pressure. The number of abdominal contractions during the 5 min test was recorded. The AWR test was employed to evaluate the abdominal reflex by inserting a lubricated silicone blister into the rectum and attached to a sterile syringe filled with water. This test allowed us to evaluate the responses to the colon-rectal distension, and in fact, each animal was assigned a score ranging from 0 to 4.

A score 0 means that no behavioral response has been observed, score 1 means that the animal remained motionless during distension in the colon or had some occasional movement, score 2 means that a weak abdominal contraction was observed, score 3 means that a strong abdominal contraction was observed, and score 4 means that the animal had violent contractions of the body and abdomen [37].

### 4.6. ELISA Kits

We used the specific ELISA kits to evaluate the concentration of Interleukin (IL)-6 (KMC0061), IL-10 (BMS614INST), IL-17 (BMS6001), and IL-23 (BMS6017) in the mouse serum according to the manufacturer’s protocols.

### 4.7. Histological Analysis

To assess colonic morphological changes, we performed the histological analysis as previously described [8]. Immediately after the animals’ sacrifice, colon tissues were attached in 10% (*w*/*v*) PBS-buffered formaldehyde solution at 25 °C for 24 h. Then the tissues were dehydrated through an increasing alcohol scale (50%, 70%, 95%, 100%, and xylene), and finally included in paraffin (Bio-Optica, Milan, Italy). Moreover, the samples have been cut via microtome to obtain 7 μm thick sections. To check morphological evaluation H&E staining (Bio-Optica, Milan, Italy) was performed, evaluating alterations like neutrophilic infiltration, edema formation, and colonic architecture. We used the following morphologic criteria for the histological analysis: score 0 no morphologic damage; score 1 focal epithelial edema as well as necrosis; score 2 diffuse inflammation and necrosis of villous area; score 3 presences of neutrophilic infiltration in submucosa area; score 4 necrosis and neutrophil infiltration; score 5 vast neutrophilic infiltrations and bleeding. All sections were examined using a Nikon Eclipse Ci-L microscope. The results of the histological investigation were displayed at 20× magnification (50 µm scale bar).

### 4.8. Immunohistochemistry Analysis of p-Selectin and ICAM-1

The immunohistochemistry analysis was performed as described before [40]. At the end of all stages, we obtained the sections to incubate all night with the primary antibody at room temperature overnight. The used antibodies were p-Selectin (Santa Cruz Biotechnology, Dallas, TX, USA, sc-8419 1:100 in PBS *v*/*v*) and ICAM-1 (Santa Cruz Biotechnology, sc-7891, 1:100 in PBVS *v*/*v*). The following day the sections were rinsed with PBS and incubated with a secondary antibody (Santa Cruz Biotechnology) for 1 h at room temperature. After incubation, the coloring agent (brown DAB) was used together with Nuclear Fast Red counter-staining. All the sections were observed with a Nikon Eclipse Ci-L microscope and analyzed at 20× and 40× magnification.

### 4.9. Immunofluorescence Analysis of the CD4, CD8 and CD68

The entire colon opened on the antimesenteric line, was included in paraffin and subsequently cut to a thickness of 7 μm via microtome. The obtained sections of 7 µm have been deparaffinized and re-hydrated to get the immunofluorescence assay as previously described [41]. Following this procedure, multiple primary antibodies were investigated, particularly the CD4 (1:100; Santa Cruz Biotechnology, Dallas, TX, USA; sc-13573), CD8 (1:100; Santa Cruz Biotechnology, Dallas, TX, USA; sc-1177), and CD68 (1:100; Santa Cruz Biotechnology, Dallas, TX, USA; sc-20060). After one night of incubation with the primary antibodies, sections were then covered with a secondary antibody—a fluorescein-isothiocyanate (FITC)-conjugated anti-mouse Alexa Fluor-488 antibody (1:2000 *v*/*v* Molecular Probes, Altrincham, UK)—for 3 h at room temperature. At the end of the 3 h, we washed the sections with PBS in a dark environment. Then, nuclei were stained by adding 2 µg/mL 40, 60-diamidino-2-phenylindole (DAPI; Hoechst, Frankfurt, Germany) in PBS. At last, the sections were observed with 20× magnification using a Nikon Eclipse Ci-L microscope to photograph the entire portion of the colon. For every antibody analyzed, positive cells were counted stereologically on sections by examining the most brightly labeled pixels and applying settings that allowed clear visualization of structural details, and while keeping the highest pixel intensities close to 200, we established contrast and brightness. The same settings were used for all images obtained from the other samples that had been processed in parallel. Digital images were cropped, and figure montages prepared using Adobe Photoshop 7.0 (Adobe Systems; Palo Alto, CA, USA) as previously reported [42].

### 4.10. Western Blot Analysis of TLR4, Myd-88, TRAF-6, NLRP3, ASC, and Caspase-1

The Western Blot analysis was completed as previously explained [43]. Briefly, after collecting the animal’s colon cytosolic and nuclear proteins were extracted. The tissue samples were suspended in two different buffers (buffer A and B) to extract the cytosolic and nuclear fractions. Buffer A contains 0.2 mM PMSF, 0.15 mM pepstatin A, 20 mM leupeptin, and 1 mM sodium orthovanadate. Subsequently, samples homogenized with buffer A were centrifuged at 12,000 rpm for 4 min at 4 °C. The supernatants obtained represented the cytosolic portion, the pellets instead represent the nuclear part. Immediately after, the pellets were suspended in buffer B and centrifuged for 10 min at 4°C, and the supernatants part was collected and stored at −20 °C for further analysis. Buffer B contains 1% Triton X-100, 150 mM NaCl, 10 mM Tris–HCl pH 7.4, 1 mM EGTA, 1 mM EDTA, 0.2 mM PMSF, 20 mM leupeptin, 0.2 mM sodium orthovanadate. Protein samples were used for the SDS page, subsequently, membranes were incubated overnight with the following primary antibodies: anti-TLR-4 (1:500 Santa Cruz Biotechnology sc-293072, Dallas, TX, USA), anti-MyD-88 (1:500 Santa Cruz Biotechnology sc-11356, Dallas, TX, USA), anti-TRAF-6 (1:500 Santa Cruz Biotechnology sc-7221, Dallas, TX, USA), anti-NLRP-3 (1:500 Invitrogen SE-2384092, Waltham, MA, USA), anti-ASC (1:500 Santa Cruz Biotechnology sc-22514, Dallas, TX, USA), and anti-Caspase-1 (1:500 Santa Cruz Biotechnology sc-56036, Dallas, TX, USA). After that, the membranes were washed and incubated for 1 h at room temperature with the relative secondary antibody (1:1000, Jackson ImmunoResearch, West Grove, PA, USA). To confirm that we used the same amount of protein sample during the procedure, we also incubated for one hour at room temperature the following primary anti-β-actin antibody (1:500; sc-47778; Santa Cruz Biotechnology, Dallas, TX, USA). Ultimately, we evaluated the signal via chemiluminescence (ECL) detection system reagent according to the manufacturer’s instructions. Relative expression of bands for every protein analyzed were imported to analysis software (Image Quant TL, v2003) and standardized to β-actin. The relative expression of the protein bands was calculated by densitometry with Bio-Rad ChemiDoc™ XRS+software.

### 4.11. Alcian Blue/PAS Staining

Alcian blue/PAS staining was executed to evaluate goblet cells in each crypt in different mouse colon samples as previously described [44]. Following the dewaxing and hydration procedure, sample sections of the colon were stained with Alcian blue/PAS following the manufacturer’s instructions (BioOptica, Milan, Italy). At last, we dehydrated the sections, with different alcohol until using xylol, and finally observed the staining. Sections were observed and acquired at 20× and 40× magnification.

### 4.12. Statistical Analysis

The experimental data are expressed as mean ± standard deviation (SD) of N observations, where N represents the number of animals studied. The statistical analysis of the obtained data was conducted with one-way and two-way ANOVA followed by the Bonferroni test for multiple comparisons. Only a *p*-value less than 0.05 was considered significant.

## 5. Conclusions

Taken together, the obtained results provide an innovative overview about the pain-relieving activity and immunomodulatory abilities of *Ulva pertusa*. These pharmacological abilities could be related to the inhibition of the TLR4 and the NLRP3 complex from which the immune-inflammatory responses have been modulated. Thus, natural therapeutic strategies targeting immune response mechanisms could represent an important turning point in the pharmacological treatment of UC patients, improving their daily activities and quality of life. Despite the promising results obtained in this study, several limitations need to be addressed. First of all, preclinical models are not always able to entirely mimic human diseases in a translational way. Particularly, in humans, various factors including genetic and environmental, contribute to the onset of colitis. Furthermore, the differences in both innate and adaptive immunity between mice and humans should be considered. In addition, since we identified gut immune cells evaluating single markers by microscopy-based methods, the use of more complex techniques such as flow cytometry or mass cytometry will be able to better validate these preliminary findings, thus providing more robust characterisation regarding the immunomodulatory activity of *Ulva pertusa*.

In light of this, future studies using gene silencing in vitro models or knockout in vivo models will be able to establish in detail the activity of *Ulva pertusa* on TLRs and specifically on TLR4 in the context of UC and other IBDs, thus evaluating its possible use as a dietary supplement.

## Data Availability

All the results were included in this study and available to the corresponding author’s address.

## Supplementary Materials

The following supporting information can be downloaded at: https://www.mdpi.com/article/10.3390/md21050298/s1, Figure S1: Assessments of body weight, colon length and histological damage following *Ulva pertusa* administration.

## Abbreviations

IBDs	inflammatory bowel diseases
CD	Crohn’s disease
UC	ulcerative colitis
GI	gastrointestinal
DNBS	2,4,6-dinitrobenzene sulphonic acid
TLR4	toll-like receptor 4
NLRP3	nucleotide-binding domain, leucine-rich–containing family, pyrin domain–containing-3
PUFA	polyunsaturated fatty acids
SIRT1	NAD-dependent deacetylase sirtuin-1
Nrf2	nuclear factor erythroid 2-related factor 2
NF-κB	nuclear Factor-κB
VMR	visceromotor response
AWR	abdominal withdrawal reflex
EMG	electromyography
IL-6	Interleukin 6
IL-10	Interleukin 10
IL-17	Interleukin 17
IL-23	Interleukin 23
PBS	phosphate buffered saline
H&E	hematoxylin/Eosin
ICAM-1	intercellular adhesion molecule 1
DAB	3,3′-diaminobenzidine
CD4	cluster of differentiation 4
CD8	cluster of differentiation 8
CD68	cluster of differentiation 68
FITC	fluorescein–isothiocyanate
DAPI	4′,6-diamidino-2-phenylindole
Myd88	myeloid differentiation primary response-88
TRAF-6	tumor necrosis factor receptor-associated factor-6
ASC	apoptosis-associated speck-like protein containing a CARD
PMSF	phenylmethanesulfonyl fluoride
Tris HCL	tris(hydroxymethyl)aminomethane hydrochloride
EGTA	ethylene glycol bis (2-aminoethyl ether)-N,N,N′,N′-tetra acetic acid
EDTA	ethylenediaminetetraacetic Acid
CRD	colorectal distension
*p*	*p*-value
PAS	periodic acid–Schiff
