# Supplementary figures and images for: New Insights into the Mechanism of Ulva pertusa on Colitis in Mice: Modulation of the Pain and Immune System
Source: Mar Drugs. 2023 May 13;21(5):298. doi: 10.3390/md21050298 (PMC10223675; doi:10.3390/md21050298)

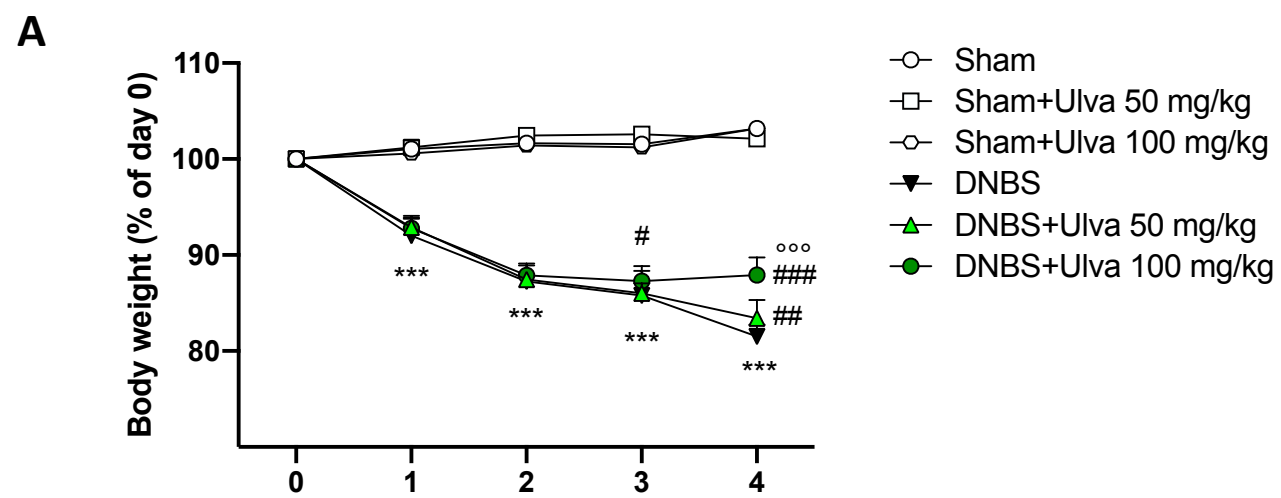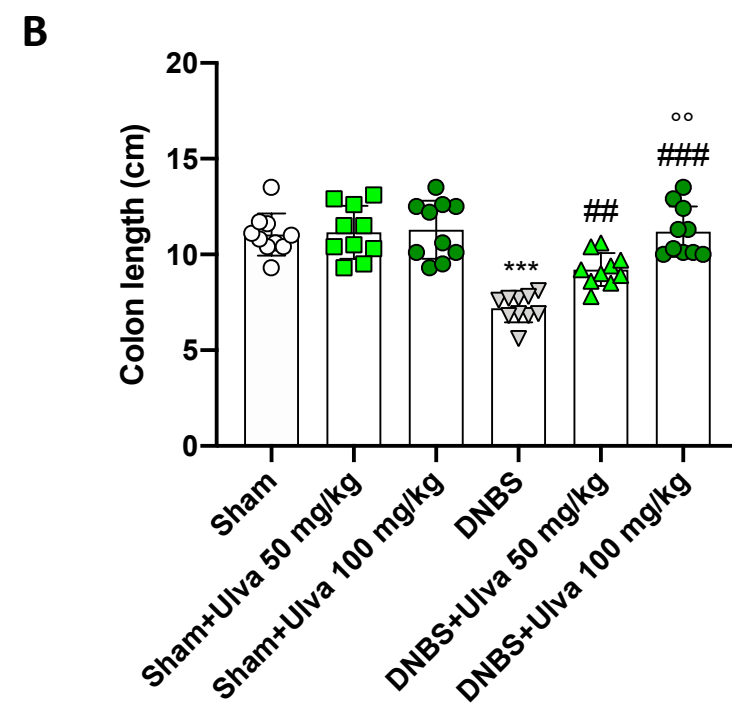

SHAM

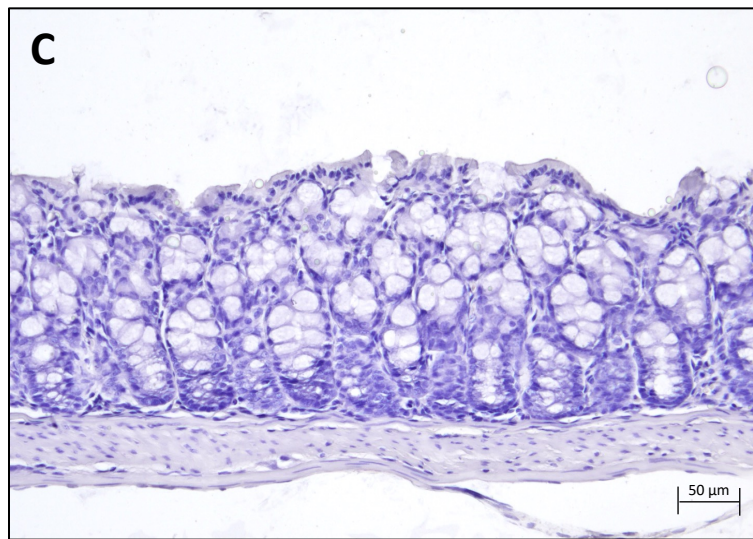

SHAM+Ulva 50 mg/kg

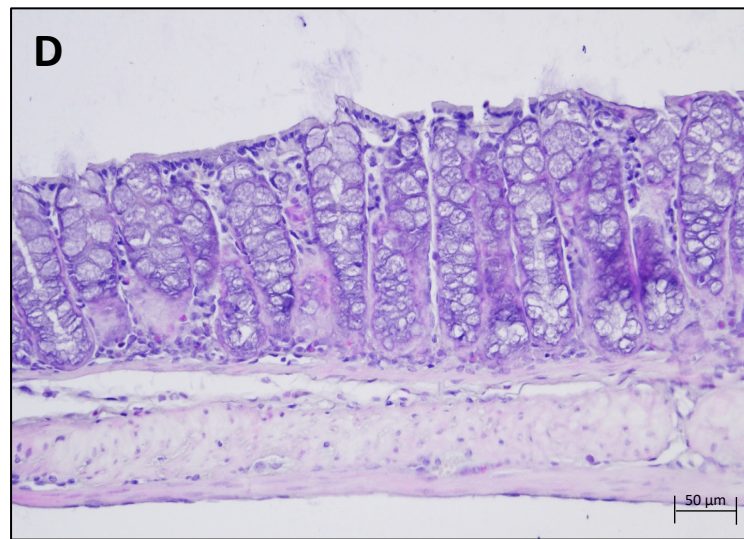

SHAM+Ulva 100 mg/kg

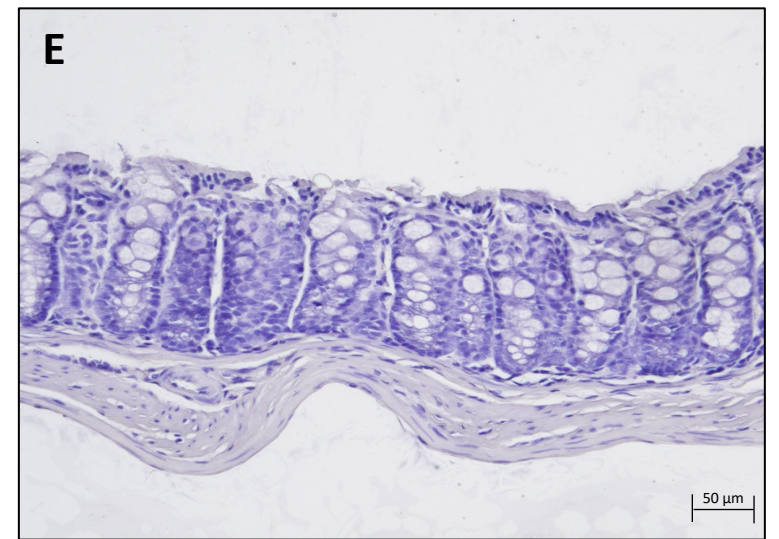

DNBS

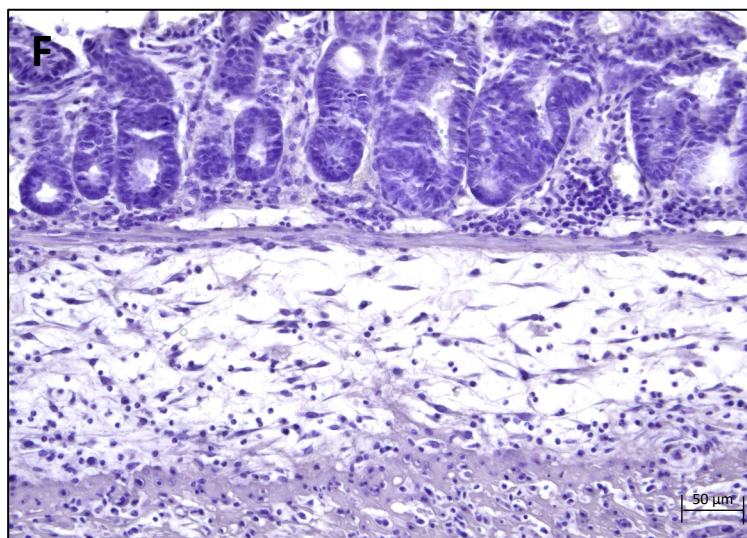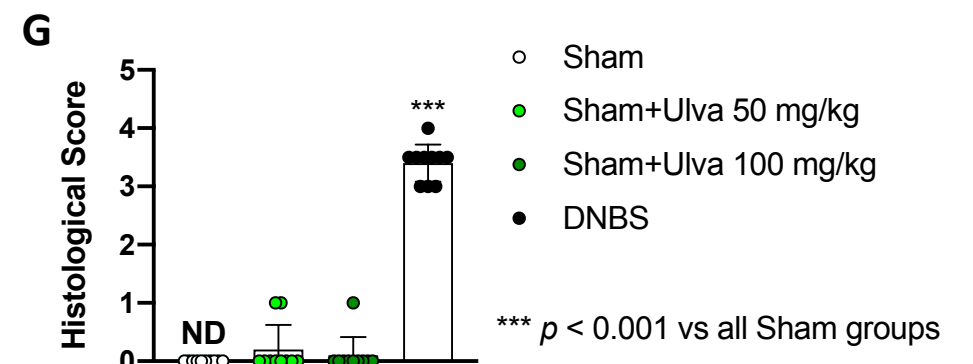

Supplement: Supplementary file 1 [file marinedrugs-21-00298-s001.zip › marinedrugs-2362217-supplementary.pdf]
